# Blockchain-based healthcare management system with two-side verifiability

**DOI:** 10.1371/journal.pone.0266916

**Published:** 2022-04-14

**Authors:** Tian Lim Tan, Iftekhar Salam, Madhusudan Singh

**Affiliations:** 1 School of Computing and Data Science, Xiamen University Malaysia, Sepang, Selangor, Malaysia; 2 Department of AI and Big Data, Endicott College of International Studies, Woosong University, Daejeon, South Korea; University College of Engineering Tindivanam, INDIA

## Abstract

The lack of data outsourcing in healthcare management systems slows down the intercommunication and information sharing between different entities. A standard solution is outsourcing the electronic health record (EHR) to a cloud service provider (CSP). The outsourcing of the EHR should be performed securely without compromising the CSP functionalities. Searchable encryption would be a viable approach to ensure the confidentiality of the data without compromising searchability and accessibility. However, most existing searchable encryption solutions use centralised architecture. These systems have trust issues as not all the CSPs are fully trusted or honest. To address these problems, we explore blockchain technology with smart contract applications to construct a decentralised system with auditable yet immutable data storage and access. First, we propose a blockchain-based searchable encryption scheme for EHR storage and updates in a decentralised fashion. The proposed scheme supports confidentiality of the outsourced EHR, keyword search functionalities, verifiability of the user and the server, storage immutability, and dynamic updates of EHRs. Next, we implement a prototype using JavaScript and Solidity on the Ethereum platform to demonstrate the practicality of the proposed solution. Finally, we compare the performance and security of the proposed scheme against existing solutions. The result indicates that the proposed scheme is practical while providing the desired security features and functional requirements.

## 1 Introduction

Ever since the beginning of the Covid-19 pandemic, the healthcare industry has been the core and focus of the world to cope with the widespread of the disease. In fact, the pandemic has exposed the weaknesses of the current centralised approach for healthcare management [1]. A common practice is to outsource the healthcare data to a cloud service provider (CSP). Sharing the health records may help improve the accuracy of disease diagnosis and promote research in the medical field. However, it possesses a high level of security concern over accessible health data as such information is considered sensitive and confidential. Searchable Symmetric Encryption (SSE) with proper key management can reduce the risk of Electronic Health Records (EHR) security compromises. Nevertheless, most proposed SSE schemes are centralised. EHRs are also hardly shareable due to the lack of verification on both sides of client and server nodes. Even though there are numerous proposed solutions for data integrity assurance, most of such solutions are not tested with implementation or applied in real life. In 2018, Hölbl, Kompara, Kamišalić, and Zlatolas [2] conducted a systematic review of the use of blockchain in the healthcare system and claimed a measurable gap between the proposed solutions and the implemented solutions year-over-year. Their work shows a large disparity between the proposed and the implemented solution. The result indicates that the research field for developing practical decentralised healthcare systems still has room for improvements.

The emergence of blockchain technology with smart contract applications created a newly found decentralised platform for auditable yet immutable data storage and data access. In recent years, several studies proposed blockchain-based decentralised data storage systems [3–9]. However, many of these proposed schemes [3, 7–9] do not support data encryption. On the other hand, some of these blockchain-based schemes incorporate encryption functionality but do not include searchability [9], or dynamic updates of the search index [4–6]. We aim to address some of these limitations in our work. We also study the system implementation practicality of such systems. In general, the scope of this paper includes healthcare management systems, blockchain technology and searchable encryption. These three elements are studied in depth to realise an efficient healthcare management system in terms of security and performance. Specifically, the interest in searchable encryption will be focused more on SSE and its implementation on a blockchain network.

We propose a practical yet secured scheme called Blockchain-based Healthcare Management System with Two-side Verifiability (BHMV) that utilises SSE and blockchain network for EHRs management. Our proposed scheme uses smart contracts on the blockchain for EHR’s index storage and the searching process. BHMV supports confidentiality of the outsourced EHR and its index, keyword search functionalities, verifiability of the user and the server, storage immutability, and dynamic updates of EHRs. All these properties are supported throughout the EHR searching and retrieving phases. In particular, to achieve storage immutability, the encrypted search index is stored in the blockchain network, whereas the encrypted EHR data is stored in the Interplanetary File System (IPFS)—a peer-to-peer cloud storage platform. Advanced Encrypt Standard (AES) is used for the encryption purpose and digital signature is used for the verification of the authorised entities. A bitmap index is implemented in BHMV that supports dynamic data updates.

A set of comprehensive simulations are conducted for performance assessment and security analysis of the proposed scheme. In terms of performance, the proposed scheme requires low developer effort to implement the system; hence, matching the objective of implementing a practical scheme for blockchain-based healthcare management system. Additionally, the encryption performance is efficient against a large set of EHR even with the simple and straightforward system design. Next, the BHMV scheme is relatively efficient during the EHR indexing phase in which the time required for indexing is approximately linear to the increasing EHR’s size. BHMV is a compact yet feature-packed scheme that can support two-side verifiability without compromising the performance and accessibility aspects of the outsourced EHR on the third-party storage. Overall, the proposed solution satisfies the desired security goals with a minimum trade-off in performance.

The rest of the paper is organised as follows: Section 1.1 and 1.2 introduce the background of SSE and blockchain. Section 2 compares the related works in the area of blockchain-based data storage. Section 3 and 4 discuss the details of the proposed solution and its implementation. Section 5 provides the security and performance analyses of the proposed scheme. Finally, Section 6 concludes the paper.

### 1.1 Overview of searchable symmetric encryption

**Fig 1:**
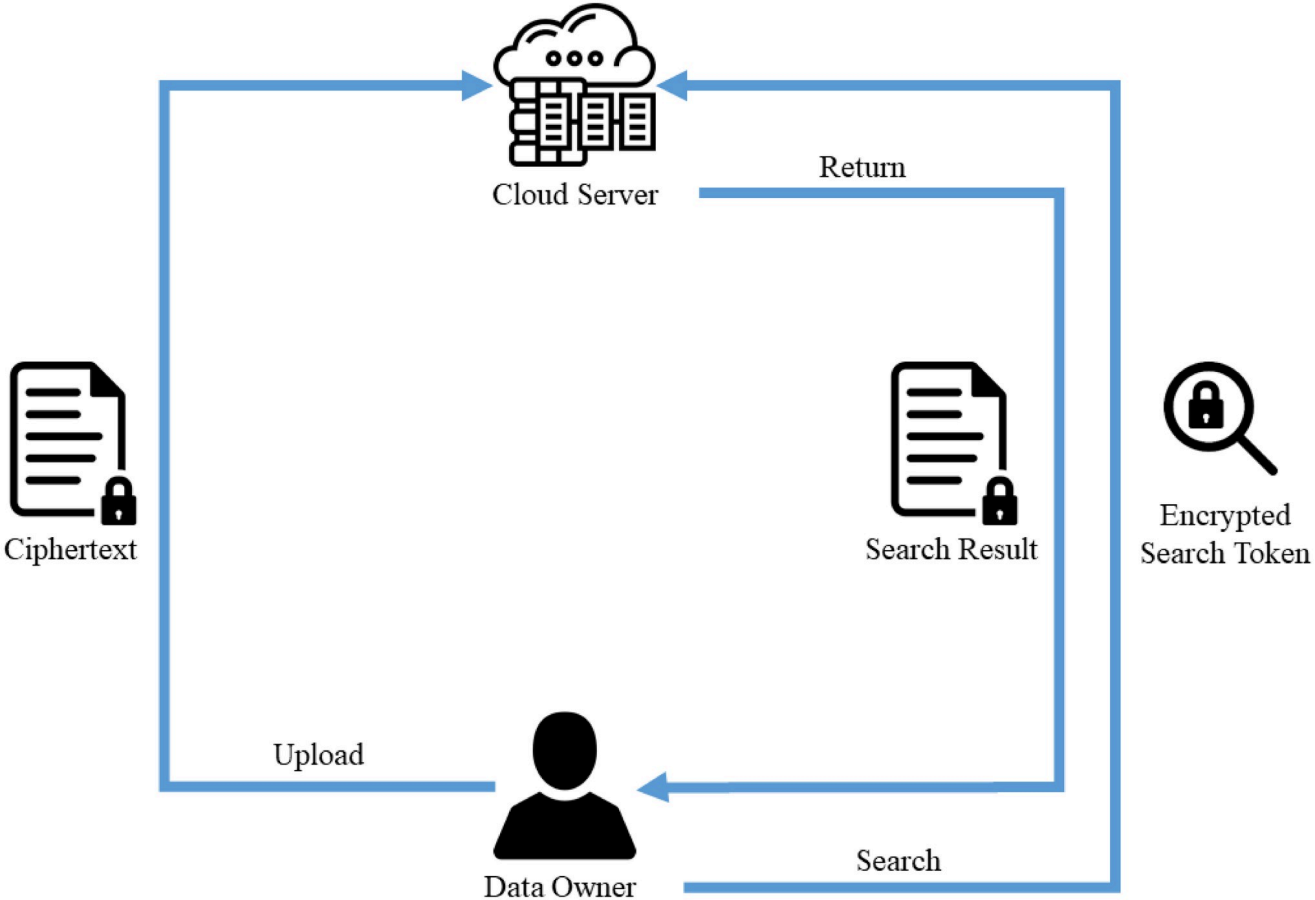
Illustration of a simple searchable encryption.

In traditional encryption, once the data is encrypted into ciphertext, it will limit the functionality and practicality of the data usage as the user cannot efficiently search or access the data. Hence, searchable encryption (SE) has been the interest to many researchers lately. SE allows users to perform a keyword search on the encrypted data by using a search token without exposing the content and search information. Fig 1 shows the overview of a basic searchable encryption scheme.

In a searchable encryption scheme:

- The data owner extracts useful information from the data before encrypting the data with the secret key.
- Constructs the index lookup table and encrypts it using the secret key.
- Upload the encrypted index and data/ciphertext to the cloud storage.
- The data owner can submit an encrypted query with a search token to search on the cloud server.
- The cloud server will perform the search process without learning the keyword and the data being searched.
- Encrypted data that satisfies the search function will be retrieved and returned back to the data owner.

According to the definition given by Curtmola et. al. [10], SSE involves mainly four polynomial-time algorithms, namely:

#### Key generation

Key generation algorithm that takes user’s security parameter *p* and returns a symmetric key *K*.

$$\begin{array}{c} KeyGen(p)\to K \end{array}$$

#### Build index

Generates an encrypted index *I* from the outsourced document *D* and the secret key *K*.

$$\begin{array}{c} IndexBuilder(D,K)\to I \end{array}$$

#### Trapdoor

Generates a trapdoor *T* as the search query by taking the generated symmetric key *K* and the user-input keyword *w*.

$$\begin{array}{c} Trapdoor(w,K)\to T \end{array}$$

#### Search

Executes at the outsourced environment to search for the document *D* that contain the keyword *w* defined in the trapdoor *T*. The search function takes the index *I* and the trapdoor *T* as the input and returns the search result *R* in the form of document pointer to the user as the output.

$$\begin{array}{c} Search(I,T)\to R \end{array}$$

The constructed index contains a pointer that can be used for locating the document after obtaining the search results. Additionally, SSE should protect the confidentiality of the outsourced documents, index, search query, search pattern and access pattern [11]. SE is more efficient than the asymmetric encryption approach because of the identical key and the underlying symmetric primitives used for encryption and decryption.

### 1.2 Overview of blockchain technology

Blockchain is formed by an open distributed ledger, in which the data is distributed and validated across the nodes based on a peer-to-peer (P2P) network [12]. The data structure in blockchain is formed by chronologically chaining the blocks of data into an irreversible chain. Every block consists of a nonce, digital signature, previous block hash and the current block hash. The hash values stored are the indicator for the order and location of the block [13].

The decentralised and distributed nature reduces the need for a trusted third party (TTP) as the participating nodes must reach a consensus for transaction verification and validation before storing it on the blockchain. There are mainly two types of consensus protocols: Proof-of-Work (PoW) and Proof-of-Stake (PoS) [14].

- **Proof-of-Work**: A block of transactions is validated by the miners competing to solve the cryptographic puzzle (hash function) by finding the random nonce and the block that holds the hash. The puzzle is considered solved if the calculated hash value of the next block starts with similar numbers of zero as the previous hash. Else, the miner will repeat the puzzle with different nonce values. The first miner who solved the puzzle will be awarded accordingly. This protocol requires immense computational power to solve the cryptographic puzzle since it is based on a trial-and-error approach.
- **Proof-of-Stake**: A block of transactions is validated by randomly chosen validators. The validators have to deposit an amount of stake in order to take part in the validation process. The validators with a higher amount of stake will have a higher chance of being chosen as the validators. The validators will be rewarded with the transaction fees inside the block and penalised if they validate a fraudulent transaction [15].

A single point of failure can be avoided as the P2P network acts as a redundant resource once any part of the system becomes unavailable. In addition, any transaction that happens on a blockchain is stored alongside immutable hash values from the previous block and the current block. A cryptographic hash function produces the hashes, and the purpose is to form a chain of the blocks that support non-repudiation and tamper-proof.

With the emergence of smart contracts, the application of blockchain extends beyond just cryptocurrency. According to Iredale [16], many industries such as supply chain, real estate and insurance are already adopting blockchain technology in their respective fields. Smart contracts allow the development of the decentralised application (Dapps) that can be deployed and used to communicate with their own private or public blockchain network.

## 2 Related work

Blockchain application research in the healthcare industry has been increasingly popular because blockchain storage can provide a durable medium for the EHR. This is because the data storage on the blockchain network is immutable, and any data modification can be easily detected by the new block created upon modification. Hence, data stored on the blockchain will subsequently become tamper-proof and support non-repudiation. In general, this section describes two different approaches of the blockchain implementation for data outsourcing.

1. Storing the entire EHR on the blockchain network.
2. Storing the EHR metadata on the blockchain network and outsourcing the EHR to third party cloud storage.

SHAREChain was first proposed in 2019 [3]. The idea behind SHAREChain is to build a private blockchain network that can ensure interoperability, reliability, as well as transparency while sharing the EHR. In this scheme, the metadata of EHR is stored on blockchain-registry and used as the index to allocate the EHR files stored in the repository. This is a relatively efficient construction, but this work did not address data privacy or confidentiality as there is no encryption deployed during data outsourcing.

Guo et al. [4] proposed a more comprehensive scheme on blockchain-based healthcare management system by incorporating Key-Policy Attribute-Based Encryption (KP-ABE). This scheme proposed a multiauthority attribute key distribution policy in which each attribute must satisfy the multiauthority predefined attribute set in order for the user to decrypt the protected file. This protocol can ensure forward security while allowing user access to be updated independently without influencing other authorised users. Even though the user access update is dynamic, the encryption for EHR is not dynamic in this scheme. Hence, the data owner cannot update or delete the encrypted EHR dynamically. Moreover, this scheme did not focus on the searchability of the encrypted EHR.

Andola et al. [5] proposed a new approach known as a secure approach for healthcare management system using blockchain (SHEMB). SHEMB uses blockchain-based SSE without involving any third party in the accessing and retrieval process. SHEMB includes three phases of process for interoperability, storage and retrieval of the encrypted EHR on the blockchain network. However, the nodes in the blockchain network such as doctors, patients, or departments might be malicious or compromised by the attackers. Therefore, user-side and server-side verifiability is an important aspect to ensure that there are no malicious activities on the pinged server or user nodes, which are not addressed in this work.

Chen et al. [6] proposed a scheme based on the five polynomial-time algorithms. The proposed solution utilises Boolean expression for extracting the keywords from the health records and constructs the index that allows the encrypted data to be searchable in the database. Apart from that, the concept of smart contract used in blockchain can guarantee payment fairness among the user nodes and miner nodes in the network. This scheme concentrates more on the searching algorithm that explores issues such as search efficiency and payment fairness. Storage enforcement and accessibility are not being thoroughly addressed in this work. Additionally, there is no actual implementation for studying the practicality of the proposed scheme.

In 2020, Asad et al. [7] proposed a permission-based blockchain healthcare data sharing scheme. The authors leveraged the implementation of Proof of Authority (PoA) algorithm to establish a permission-based access control where the data owner can dictate who and which permission levels are allowed. However, storing the whole EHR on the blockchain is not the ideal case as the block size is limited and it will incur additional cost if the storage has to be partitioned into multiple transactions in order to accommodate the EHR. Furthermore, the authors did not focus on the confidentiality and integrity of the outsourced data.

Parameswari and Mandadi [8] proposed a healthcare data protection scheme leveraging blockchain technology. This scheme lacks confidentiality and the integrity of the EHR. Neither encryption scheme nor verification algorithm is used during the outsourcing of the EHR. The EHR storage approach is the same as the scheme proposed in [7], which is inefficient due to the high transaction fees incurred.

Chelladurai and Pandian’s [9] scheme approaches the topic with a different implementation model. The scheme is composed of four different models: (i) Immutable log creation model, (ii) Patient-provider permission model for EHR updates, (iii) Data sharing model, (iv) Viewership model. Immutable log creation model is obligated for creating new EHR data entry and the EHR update model handles the new changes or modification of the EHR. The data sharing and viewership models control the user’s access to the EHR. The authors provide an in-depth look at the application and implementation of blockchain technology in the healthcare day-to-day operation. The proposed scheme supports dynamic EHR updates. However, this scheme does not address the encryption and keyword search on the encrypted EHR. Moreover, the permission model in this scheme lacks entity verification.

We emphasize that several other works propose schemes [17–23] that address different security aspects in electronic health record sharing. Some of these works focus only on sharing encrypted healthcare data, some examine secure communication for the healthcare system, and others focus on the authentication scheme for sharing healthcare records. Although these are interesting works on securing different aspects of healthcare records, these are not within the scope of blockchain-based searchable encryption, which is the core of this paper. Therefore, we limit the analyses presented in this paper to only blockchain-based searchable encryption.

Table 1 summarises and compares the different attributes between the studied schemes. All schemes achieved a certain level of security in EHR storage, as the distributed ledger on blockchain natively supports some security features. Even so, in order to fully protect the confidentiality of the EHR from any active or passive attacks, an encryption scheme should be implemented before the transmission of the EHR. Additionally, storing the entire EHR on the blockchain is inefficient due to the limited block size and high cost for adding a new block. Blockchain technology should be implemented as the procedure for protecting the EHR instead of the storage for the EHR. For EHR update operation, only the solution proposed by Chelladurai and Pandian [9] mentioned about dynamic data updates. However, the scheme does not apply encryption algorithms during EHR outsourcing. Hence, the dynamic data update feature in this literature is irrelevant in the context of searchable encryption.

**Table 1:** Multiuser SSE—Key features comparison (✔: Supported; ✘: Not supported).

Ref.	Encryption	Storage utilisation	Searchability	Update
[3]	✘	Metadata on blockchain EHR on repository	✔	✘
[4]	✔	Encrypted index on blockchain EHR on cloud storage	✔	✘
[5]	✔	Entire EHR on blockchain	✔	✘
[6]	✔	Encrypted index on blockchain EHR on cloud storage	✔	✘
[7]	✘	Entire EHR on blockchain	✔	✘
[8]	✘	Entire EHR on blockchain	✔	✘
[9]	✘	EHR’s hashes on blockchain EHR on databases	✘	✔

## 3 Proposed solution

We introduce the model of the proposed scheme, Blockchain-based Healthcare Management System with Two-side Verifiability (BHMV). In the below sections, we discuss the functions and methods used in BHMV.

### 3.1 System model

In BHMV, we mainly focus on the implementation of Searchable Symmetric Encryption (SSE) in the healthcare management sector to secure the confidentiality of the Electronic Health Record (EHR) without compromising its usability and accessibility. For enabling searchability over encrypted EHR, a bitmap index is constructed in the data pre-processing stage. The searchable encryption implemented in BHMV uses the Cipher-Block Chaining (CBC) encryption mode of the Advanced Encryption Standard (AES) algorithm.

To realise two-side verifiability, Elliptic Curve Digital Signature Algorithm (ECDSA) is used to safeguard the authenticity of the searched query or returned result. However, ECDSA requires a digitally signed certificate issued by a Trusted Third Party (TTP) or a Certificate Authority (CA). The purpose of the digital certificate is to vouch for the validity of the ECDSA public key. Generally, the certificate contains the user’s public key, user ID, the certificate issue date and the expiration date. This approach is not optimal as it increases the degree of point of failure since the TTP or CA might be a constant primary target for the attacker to compromise the whole system. Furthermore, the renewal or revocation of a certificate is relatively inefficient if the system is required to process a large number of datasets [24].

To limit the number of entities involved in the system, the TTP is replaced by a blockchain network such as Bitcoin or Ethereum. Authorised users can share their ECDSA public keys using the smart contracts on the blockchain. Modifying any block of the data on the blockchain will not overwrite the previous data. Instead, a new block is being mined and chained to the previous block. Any metadata such as account address, timestamp, and previous hash value will be stored in the new block. Therefore, any malicious attempt carried out by the attacker will be recorded in the blockchain forever. As a result, the details of the attacker are traceable for further investigation or access blacklisting. Hence, the ECDSA public key is immutable from intentional corruption and the recorded malicious block can be used as the proof of attack against the attacker’s blockchain account.

Additionally, BHMV is using the blockchain distributed ledger to store the encrypted bitmap index and verify the search requests from the authorised user. Blockchain storage can ensure the integrity of the index and consequently ensure the search result to be accurate. The returned search result is also authenticated using the ECDSA signature.

#### 3.1.1 The bitmap index

In our proposed scheme, all the uploaded EHRs will be indexed before encryption takes place. A bitmap index is implemented in BHMV to store the extracted keyword with its corresponding EHR unique pointer or identifier. A bitmap index is an index matrix that allows the system to search among the encrypted keywords and locate the EHR. The extracted keyword represents the row key of the matrix while the EHR identifier represents the column key of the matrix. The values stored are Boolean values, consists of only “0” and “1”; where “0” stands for the absence of a particular keyword in a particular EHR while “1” represents the presence of the keyword. As illustrated in Table 2, if the keyword “recovery” is present in EHR_001, the value of the corresponding matrix cell will be “1”. Table 2 illustrates an example, where during the event of updating the bitmap index, the EHR owner only needs to update the Boolean value at the corresponding row. For example, consider that an update of the EHR, the keyword “recovery” is now also present in EHR_003. Hence, the EHR owner only needs to update the index of the keyword to 1011.

**Table 2:** Illustration of the bitmap index.

Keyword	EHR_ID			
	EHR_001	EHR_002	EHR_003	EHR_004
recovery	1	0	0	1
cancer	0	0	0	1

#### 3.1.2 Smart contract

A smart contract is a self-executable program that can be deployed on the blockchain network. In fact, the smart contract is firstly used for the transaction of cryptocurrencies, a newly found digital representation of the monetary system. Transaction will be executed automatically when a predefined set of conditions are met. The execution of transactions via smart contract is reliable because all the transactions are recorded and irreversible. In our scheme construction, a smart contract is used for uploading and searching the bitmap index aforementioned. The smart contract used for index searching also supports ECDSA digital signature verification to prevent any malicious party from gaining access or altering the search token and its corresponding result. To reduce the single point of failure in the scheme, blockchain implementation can minimise the entities involved in the system.

#### 3.1.3 Entities in BHMV

**Fig 2:**
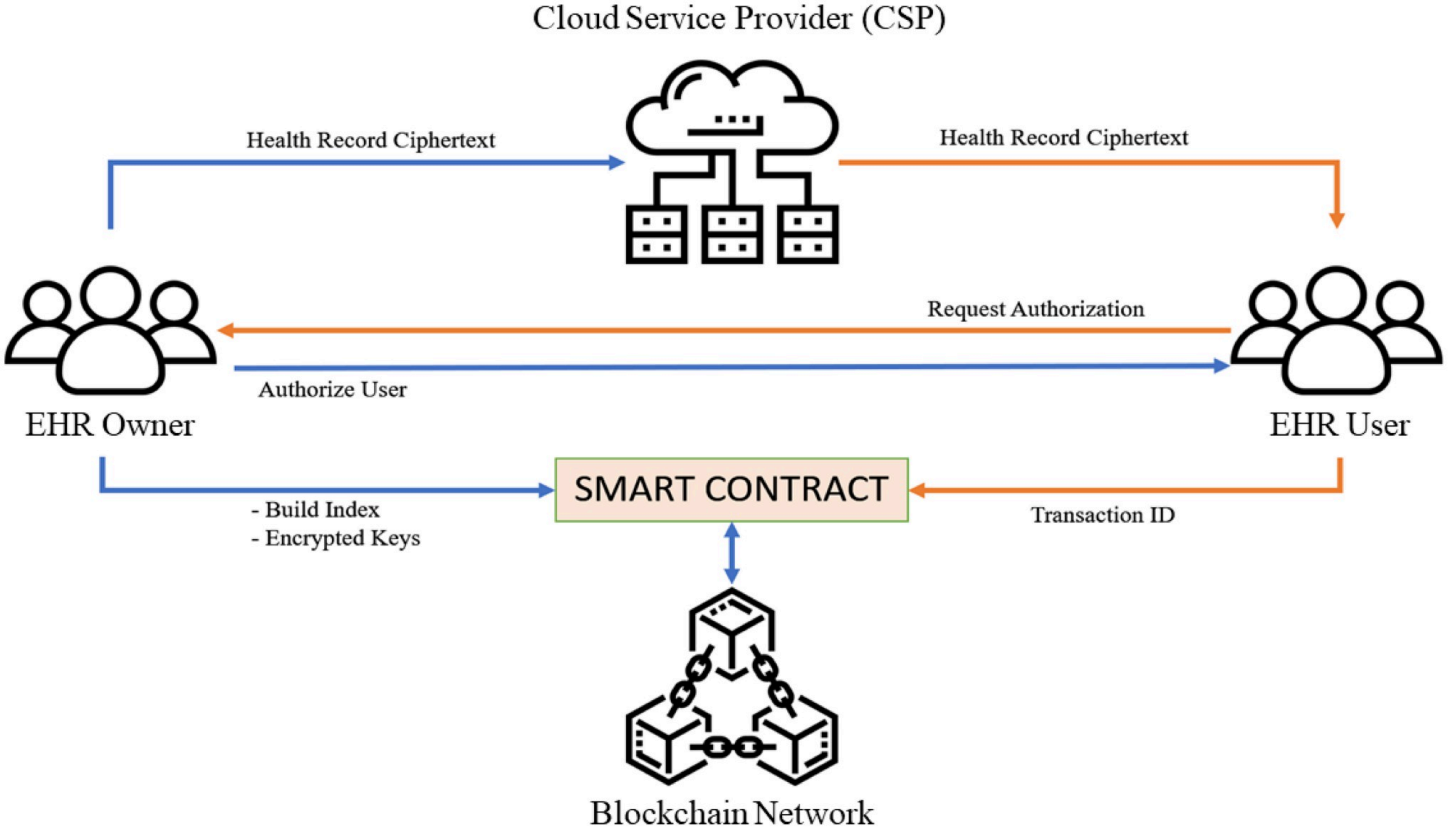
Theoretical framework of the proposed BHMV model.

The framework of the proposed BHMV scheme is depicted in Fig 2. There are mainly four entities involved in the use case, namely the EHR owner, the EHR user, the cloud service provider (CSP) and the blockchain network. In a single user use case, the EHR owner and the EHR user are the same entity.

- **EHR owner**: The entity that holds the original data of EHR. The EHR owner is responsible for extracting the keywords and building the corresponding index from the EHR. After that, the owner will encrypt the EHR and its index and upload them to the cloud storage and blockchain network, respectively. The smart contracts used in the blockchain must be deployed by the owner beforehand.
- **EHR user**: The entity that generates the search token as a query to search and access the EHR. EHR users must be authorised by the EHR owner unless the EHR owner and EHR user are the same entity.
- **Cloud service provider (CSP)**: This entity is responsible for storing the encrypted EHR that is outsourced by the EHR owner. The search result obtained is used to retrieve the EHR from the cloud service provider.
- **Blockchain network**: Smart contract is used for public key management and index searching. The index is uploaded by the EHR owner and stored safely inside the blockchain as a block of data. Any entity who wishes to search the EHR needs to get access information from the smart contract owner, i.e., the EHR owner.

### 3.2 Definition of BHMV

The proposed model of BHMV consists of five stages of operation: System Setup, EHR Pre-processing, EHR Storage, EHR Searching and Access, and Access Revocation.

- **System setup**: This stage initialises all the components or parameters required before the EHR is being outsourced to the CSP. First of all, the EHR owner initiates the key generator to generate a pair of secret keys (*K*_1_, *K*_2_) for EHR encryption in the data storage stage. EHR owner and users generate a pair of ECDSA public and private keys (*PK*_*U*_, *SK*_*U*_) for the usage of entity authentication. At the same time, CSP will also generate a pair of ECDSA public and private keys (*PK*_*S*_, *SK*_*S*_) for authentication purposes.
- **EHR pre-processing**: EHR owner has to build an index and encrypt the EHR before uploading it to the offshore storage space. In this stage. the owner extracts the keywords set from each EHR and indexes the EHR accordingly so that the EHR user can search for the EHR later on in the searching stage. The index in this scheme is created using the bitmap index as it can efficiently support dynamic data updates. Note that the bitmap index built in this stage is temporary and not in the final form yet.
- **EHR storage**: The owner encrypts the EHR with the symmetric key *K*_1_ and then uploads the EHR ciphertext to Interplanetary File System (IPFS), which is a peer-to-peer cloud storage platform. The bitmap index is encrypted using another symmetric key *K*_2_ and uploaded to the blockchain for immutable storage.
- **EHR searching and access**: The authorised users have the authority to access the blockchain network and use the smart contract to search the particular keyword they are interested in. The search token generated needs to be authenticated with the digital signature before searching the bitmap index matrix. The returned result requires to pass the authentication test to realise the two-side verification process proposed in this scheme.
- **Access revocation**: In the case of revoking user access from EHR, the data owner deletes the user’s ECDSA public key pair from the blockchain. Since the search token requires ECDSA authentication, the search token from the revoked user will fail the test. As a result, the revoked user loses access to the EHR and the index stored on the blockchain.

### 3.3 BMHV system walkthrough

**Fig 3:**
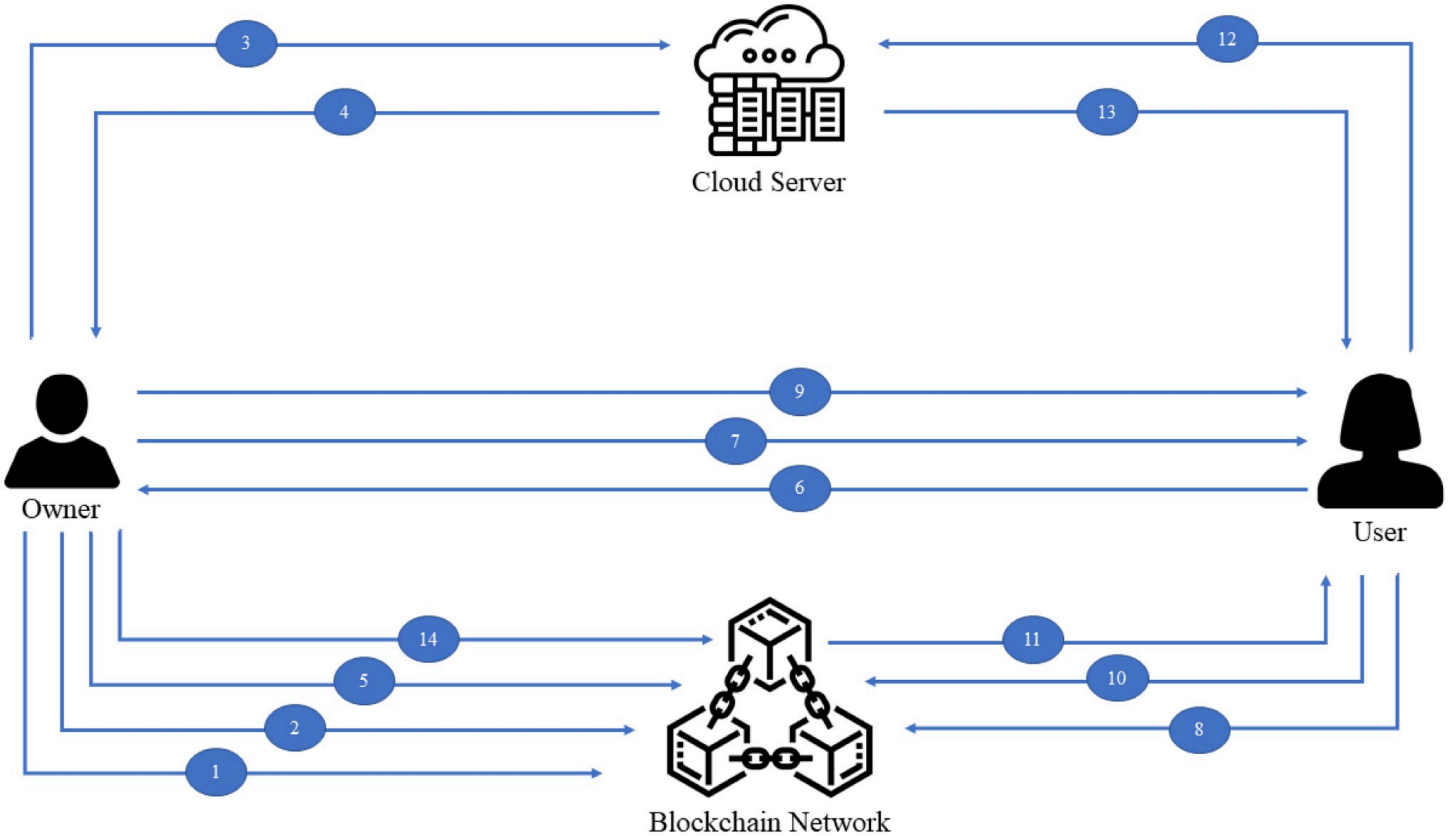
The working process of BHMV.

The processes involved in the proposed scheme are illustrated in Fig 3. Explanation of the processes is given in chronological order according to the different steps involved. Step 1 and Step 2 are associated with the system setup process, while Step 3 to Step 5 are associated with the EHR pre-processing and storage processes. Step 6 to Step 13 illustrate the EHR searching and access process. Lastly, Step 14 is related to the access revocation process. IPFS is used as the cloud storage server in the proposed model. We discuss these steps as follows:

- Step 1: The EHR owner compiles and migrates the smart contracts to the blockchain and receives the contract address and Application Binary Interface (ABI) for access usage.
- Step 2: The EHR owner uploads the established ECDSA public keys to the blockchain for sharing and ensuring the integrity of the public key.
- Step 3: After indexing the EHR, the owner encrypts the EHR with the symmetric key *K*_1_ and uploads the EHR ciphertexts to the IPFS.
- Step 4: The hash value from IPFS is recorded as the identifiers for each EHR. Hence, the temporary bitmap index from the previous stage will be updated by replacing the matrix’s column with the file hash.
- Step 5: The bitmap index is encrypted using the other symmetric key *K*_2_ and uploaded to the blockchain for immutable storage.
- Step 6: The EHR user acquires permission from the EHR owner by sending an access request to the owner alongside his or her ECDSA public key.
- Step 7: If the owner approves the request, the smart contract address and ABI will be sent to the user.
- Step 8: The EHR owner stores the approved user’s ECDSA public key in the authorised user control list via smart contract.
- Step 9: The owner will then retrieve the user’s ECDSA public key and encrypt the EHR and index’s symmetric keys using the user’s ECDSA public key. Then, the owner sends the symmetric keys ciphertext to the user. The EHR user decrypts the received ciphertext with his or her ECDSA private key to obtain the symmetric keys (*K*_1_, *K*_2_) used for search token generation and EHR decryption.
- Step 10: The user inputs the keyword to the token generator to generate an encrypted search token that can be used for searching the smart contract. However, for ensuring the search query is generated from a legitimate user, an ECDSA-based digital signature is used to authenticate the originality of the search token. ECDSA is also used for user access revocation in Step 14. The user hashes and signs the search token with his own ECDSA private key *SK*_*U*_. Next, send the search token alongside the digital signature to the blockchain and the smart contract will verify the query using the user’s public key *PK*_*U*_ that is stored in the blockchain during the access request stage. If the output is valid, the smart contract proceeds to search the encrypted index for the matching token pair.
- Step 11: The search result will be returned in the form of a file hash.
- Step 12: After that, the user retrieves the EHR ciphertext using the hash value returned from the search function.
- Step 13: The integrity of the returned EHR can be ensured because if the EHR has been unintentionally or intentionally modified, the file hash will be updated and different from what the owner has stored on the blockchain. As a result, a modified EHR can be detected by comparing the hash value on blockchain and the hash value on IPFS. Finally, the EHR user decrypts the ciphertext with the symmetric key *K*_1_.
- Step 14: Data owner removes (updates the smart contract) user’s ECDSA public key pair from the authorised user list on blockchain.

## 4 Concrete construction of BHMV

This section provides a detailed look at the construction of the Blockchain-based Healthcare Management System with Two-side Verifiability (BHMV). Sect. 4.1 introduces the notations and symbols used to describe our construction. Sect. 4.2 discusses the operations involved in system setup; while Sect. 4.3 elaborates the electronic healthcare record (EHR) pre-processing. Next, Sect. 4.4 describes the EHR storage in an outsourced environment. Sect. 4.5 discusses the specific design and algorithms used for EHR searching and access. Then, Sect. 4.6 provides technical details on the verification of the digital signatures. Finally, Sect. 4.7 discusses the implementation details.

### 4.1 Notations and symbols

A list of all the notations and symbols used in this paper with its corresponding description is provided below.

- *KeyGen*: Cryptographic key generator
- *K*_1_, *K*_2_: Searchable symmetric encryption keys
- *p*_1_, *p*_2_: Randomly generated passphrases
- *iv*: Initialisation vector
- *ENC*: Searchable symmetric encryption
- *DEC*: Searchable symmetric decryption
- *PK*_*U*_, *SK*_*U*_: User’s public and secret key
- *PK*_*S*_, *SK*_*S*_: Server’s ECDSA public and secret key
- *M*: Bitmap index matrix
- *c*_*ij*_: Matrix’s cell at row *i* column *j*
- *h*_*j*_: File hash value at column *j* in the bitmap matrix
- *w*: Encrypted keyword
- *W*: Encrypted keywords set stored on the Blockchain
- *C*_*EHR*_: EHR ciphertext
- *C*_*M*_: Bitmap index matrix ciphertext
- *H*: Hash function
- *HASH*: Hash value output from the hash function
- *SIGN*: ECDSA signature function
- *T*_*w*_: Search token
- *Tag*: ECDSA signature/verification tag
- *R*: Search result
- *ECREC*: ECDSA signature recovery function
- *Rec*_*Tag*_: Recovered value of ECDSA signature
- *ε*: An empty string

### 4.2 System setup

Since searchable symmetric encryption is used for preserving EHR’s confidentiality, the EHR owner initialises the security parameters as two randomly generated passphrases. Next, the owner passes the passphrases into the key generator for generating two unique symmetric encryption keys. Two different symmetric keys are assigned for EHR file encryption and index encryption, respectively.

$$\begin{array}{c} KeyGen_{ENC}\left(p_{1},p_{2}\right)\to \left(K_{1},K_{2}\right) \end{array}$$

For realising two-side verification, EHR owner and cloud service provider (CSP) generate their pair of Elliptic Curve Digital Signature Algorithm (ECDSA) public and private key and initialise a Keccak-256 hash function.

$$\begin{array}{c} KeyGen_{ECDSA}\left(User\right)\to \left(PK_{U},SK_{U}\right)\\ KeyGen_{ECDSA}\left(CSP\right)\to \left(PK_{S},SK_{S}\right) \end{array}$$

After initialising all the required security parameters, the EHR owner will migrate and deploy the smart contracts on the Ethereum blockchain network. The owner will in turn receive the smart contracts metadata that is required to access and communicate with the contracts on the blockchain network.

### 4.3 EHR pre-processing

To realise searchability, the EHR has to be indexed first before being encrypted and outsourced to a third-party storage. EHR owner generates the index according to the steps shown in Algorithm 1. Firstly, the keyword extractor will be initialised and configured based on different criteria such as the input text language and the removal of digits and the stop word set. After the configuration, the tokeniser scan and tokenise the EHR content into a collection of discrete text strings known as token. After that, the keyword extractor filters the unnecessary tokens by comparing the token with the pre-configured stop word set. The stop word set consists of common texts, clauses or numbers that are not necessary for indexing the EHR.

**Algorithm 1**: BuildBitmapIndex

1 Initialise index writer

2 Configure the analyser for the index writer

3 Initialise a matrix *M* for bitmap index

4 **for** *each EHR* **do**

5  Breakdown the text field into tokens

6  Extract and index the token

7  Attach keyword *w* to the matrix row

8  Attach file hash *h*_*j*_ to the matrix column

9  **if** *w*_*i*_ ∈ *EHR*_*j*_ **then**

10   cell *c*_*ij*_ in *M* = 1

11  **else**

12   cell *c*_*ij*_ in *M* = 0

13 **return** *M*

Next, Algorithm 1 initialises a two-dimensional array as a matrix for storing the information about the EHR’s index. The encrypted keywords are attached to the first column of the matrix; while the hash value of the EHR returned by the Interplanetary File System (IPFS) storage is attached to the column of the matrix, representing the column identifier. Lastly, the algorithm will check the presence of each keyword in the particular EHR and fill the cells with “1” if the keyword is present in the file; otherwise, it will remain “0”.

### 4.4 EHR storage

AES-CBC mode is used as the underlying encryption algorithm. Therefore, the EHR owner generates an initialisation vector *iv* as the input for the encryption to prevent the repetition of ciphertext generated from the similar plaintext encryption. The *iv* generated consists of 16 bytes of randomised block. CryptoJS library is used to generate the *iv* as well as the 256-bit symmetric keys *K*_1_, *K*_2_. Next, the EHR owner encrypts the EHR with the *iv* and the symmetric key *K*_1_ generated during the system setup phase. The application converts the EHR to Uint8Array buffers and passes the file buffers to the encryption function for encryption. The encryption function encrypts the EHR buffers with the *iv* and *K*_1_. The ciphertext is generated in word arrays format and the word arrays are converted back to Uint8Array buffers before returning to the application. After the EHR encryption, the owner uploads the EHR ciphertext *C*_*EHR*_ to IPFS for storage outsourcing. IPFS returns the file hash as the EHR identifier. The EHR owner completes the construction of bitmap index *M* by inserting the file hash to the first row as the document identifier. The keyword encryption encrypts the keywords with another symmetric key *K*_2_. The keywords inside the bitmap index are encrypted one by one by looping through the keyword’s arrays. That is, the encryption of the EHR and the bitmap matrix are computed as:

$$\begin{array}{c} ENC_{K_{1}}\left(iv,EHR\right)\to C_{EHR}\\ ENC_{K_{2}}\left(iv,M\right)\to C_{M} \end{array}$$

Next, the owner accesses the Ethereum blockchain network by smart contract using the contract address and Application Binary Interface (ABI). The EHR ciphertext *C*_*EHR*_ is uploaded to the IPFS. The bitmap index ciphertext *C*_*M*_ is then uploaded to the distributed ledger on the blockchain.

### 4.5 EHR searching and access

In a single-user use case, the owner and the user of the EHR are the same entity. In a multi-user use case, the EHR user has to obtain permission and authorisation from the EHR owner before searching and accessing the outsourced EHR data. For searching the encrypted bitmap index *C*_*M*_ on the blockchain, the user needs to generate a search token first. Firstly, the user enters a keyword *w* to search the EHRs containing the keyword *w*. The steps of search token generation are illustrated in Algorithm 2. The algorithm encrypts the query keyword with secret key *K*_2_ to generate the search token. Next, the search token is hashed with the Keccak256 hash function to generate a fixed length of 64-byte hash. The algorithm generates the search token tag *Tag*_*w*_ by signing the output hash value with the user’s ECDSA secret key *SK*_*U*_. Lastly, the search token *T*_*w*_ and search token tag *Tag*_*w*_ are returned to the user.

**Algorithm 2**: Search Token Generation

**Input**: The query keyword *w*, EHR encryption key *K*_2_, and user’s ECDSA secret key *SK*_*U*_

**Output**: Search token *T*_*w*_ and search token tag *Tag*_*w*_

1 Initialise *T*_*w*_ = *ε* and *Tag*_*w*_ = *ε*

2 $T_{w}\leftarrow ENC_{K_{2}}\left(iv,w\right)$

3 *HASH*_*w*_ ← *H*_*Keccak*256_(*T*_*w*_)

4 $Tag_{w}\leftarrow SIGN_{SK_{U}}\left(HASH_{w}\right)$

5 **return** *T*_*w*_ and *Tag*_*w*_

After obtaining the search token, the user accesses the search function in the smart contract to query the EHR index stored on the blockchain. The smart contract will verify the validity of the search token signature first before initiating the index search function. Only search tokens from valid or authorised users can be used to search the index and return the corresponding result. The steps for index searching are illustrated in Algorithm 3. The algorithm initialises an empty array *R* for storing the search result temporarily. If the search token signature *Tag*_*w*_ is valid, the algorithm will compare the search token *T*_*w*_ to the encrypted keyword *w* in the bitmap index *M*. If the search token *T*_*w*_ appears in the list of encrypted keywords set *W*, the index searching module iterates the bitmap index at that particular keyword row to identify which EHR contains the searched keyword. If the value for a particular cell *c*_*ij*_ is “1”, the corresponding column identifier value is added to the array of search result *R*. At the end of the searching, the smart contract will hash and sign the search result *R* to realise the two-side verifiability.

**Algorithm 3**: IndexSearch

**Input**: Encrypted bitmap index *C*_*M*_, search token *T*_*w*_ and tag token *Tag*_*w*_ generated by the EHR user

**Output**: Search result *R* and search result tag *Tag*_*R*_

1 Verify the validity of *T*_*w*_ using *Tag*_*w*_

2 Initialise an empty array for storing *R* and *Tag*_*R*_

3 **if** *valid* **then**

4  **for** *each keyword w in keywords set W* **do**

5   **if** $T_{w}==C_{M_{ij}}$ *and cell c*_*ij*_ == 1 **then**

6    set *R* ← *R*∪*h*_*j*_

7  *HASH*_*R*_ ← *H*_*Keccak*256_(*R*)

8  $Tag_{R}\leftarrow SIGN_{SK_{S}}\left(HASH_{R}\right)$

9 **return** *R* and *Tag*_*R*_

### 4.6 Verification

For verifying the authenticity of the search token signature and search result signature, we use the ECDSA verification function. Algorithm 4 demonstrates the steps in the verification function for achieving the purpose of message origin validation. At first, the algorithm hashes the input message *m* and stores it as *HASH*_*m*_. Next, it uses the hash output and the signature as the parameter for the elliptic curve recovery function ECREC. The ECREC function is the class function provided in the Solidity cryptographic library. The purpose of using this function is to recover the signature’s origin public address in order to verify whether the message signer is administered inside the user access list. The recovered value returned from the ECREC function is compared with the sender’s public key/address for verification. The verification function will return true (1 or valid) if matches and proceed to search or EHR decryption depending on the use case. On the contrary, the verification function will return false (0 or invalid) if the value returned does not match, reject the search token or the search result.

**Algorithm 4**: Verification

**Input**: The input message *m* (search token *T*_*w*_ or search result *R*) and the corresponding message tag *Tag*_*m*_ (*Tag*_*w*_/*Tag*_*R*_)

**Output**: Boolean value of true or false

1 Initialise recovered signature variable *Rec*_*Tag*_

2 *HASH*_*m*_ ← *H*_*Keccak*256_(*m*)

3 *Rec*_*Tag*_ ← *ECREC*(*HASH*_*m*_, *Tag*_*m*_)

4 **if** *Rec*_*Tag*_ == *sender’s address* **then**

5  **return** true

6 **else**

7  **return** false

### 4.7 Implementation of BHMV

The proposed BHMV scheme is implemented on a Windows 10 (64-bit) machine with an Intel Core i5-7300HQ processor, NVIDIA GeForce GTX 1050 graphic card, and 8 gigabytes (GBs) of random-access memory (RAM). The programming languages used for the implementation are JavaScript, Hypertext Markup Language (HTML), and Solidity.

#### 4.7.1 Development environment and libraries

**Fig 4:**
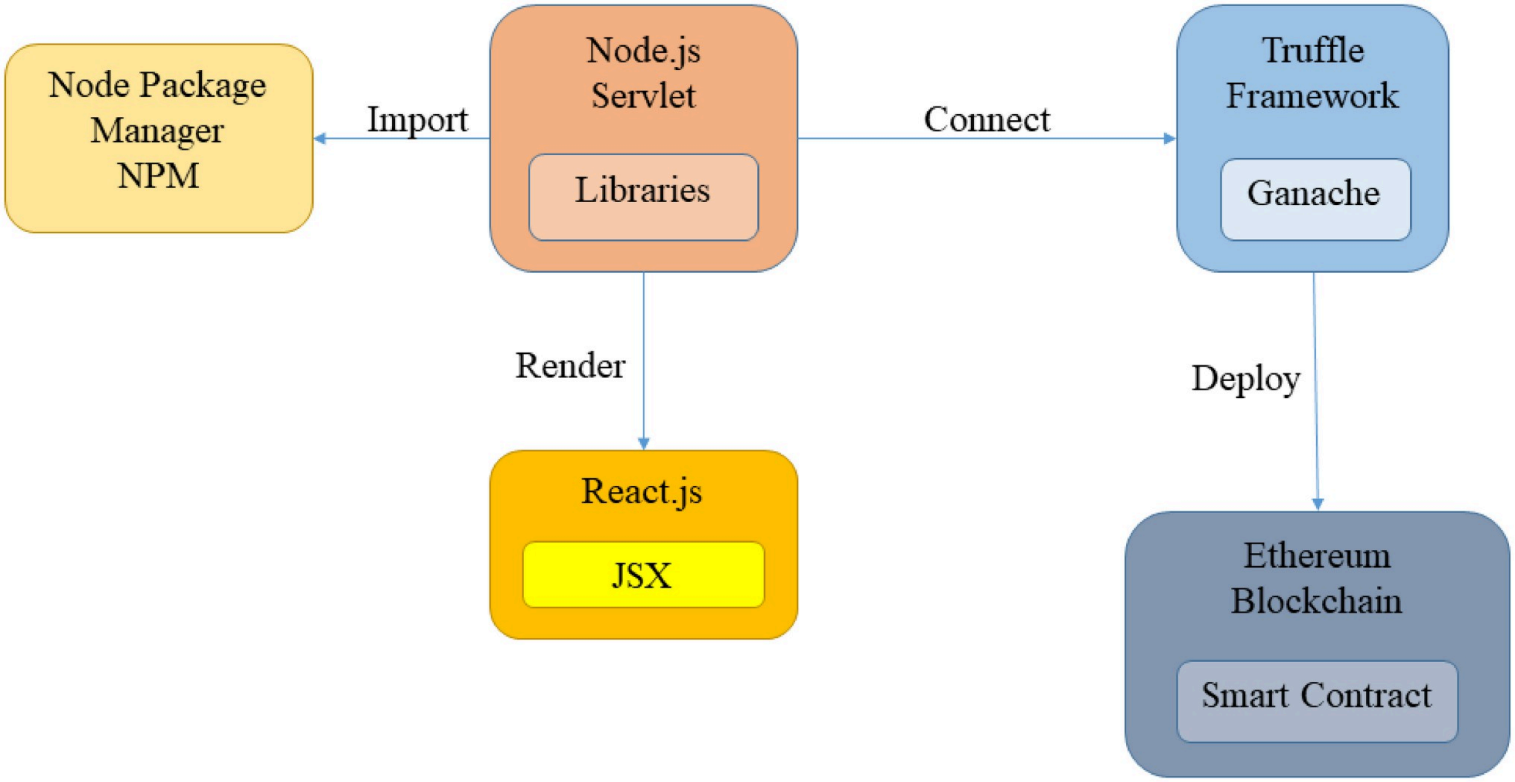
High-level overview of the development environment.

The environment is set up using Node.js, which is a run-time environment for compiling and executing the written JavaScript programs. Fig 4 illustrates the high-level overview of the components involved in the development environment. In this architecture, Node.js plays the role of back-end framework in the project as most of the scripts are rendered on the Node.js server environment before transmitting to the web browser for front-end user interaction. Speaking of front-end development, React library is imported and JavaScript XML (JSX) syntax extension is used for developing the front-end user interfaces. React can translate the written JSX script to regular JavaScript at runtime, allowing the developers to write HTML code in React components without using extra methods such as createElement() and appendChild(). The Node.js environment is developed using IntelliJ IDEA Integrated Development Environment (IDE).

The libraries used in the project are installed using Node Package Manager (NPM) in the form of an NPM registry. NPM is an open-source platform for software distribution and dependencies management. For EHR storage outsourcing, IPFS is used as the peer-to-peer distributed storage solution. IPFS hashes the uploaded EHR and stores the hash values using a distributed hash table (DHT). Then, IPFS distributes the hash keys to participating peers’ nodes on the distributed network. In the proposed scheme, the hash keys are stored on the blockchain as the EHR index, making the hash keys immutable and traceable for any malicious modification attempt. Ethereum is used as the medium for the blockchain network implementation in BHMV. Our scheme develops the smart contracts using Remix IDE with Solidity programming language. The smart contracts are compiled and deployed using the Truffle Suite framework.

Dummy Ethereum accounts with free test Ether (ETH) cryptocurrency are created using the Ganache component provided inside the Truffle Suite package. Ether is the virtual currency used in Ethereum for the transaction to monetise the miner nodes. Ganache is a personal blockchain network that supports efficient Ethereum decentralised application development. Each account is credited with 100 test ETH and the address is the account identifier, also the public key for digital signature verification during the Ethereum transaction. To spend and use the Ether coin, Metamask cryptocurrency wallet is used to finance the transaction. The users can connect their account with the Metamask wallet using the Ethereum account private key. Whenever the owner wants to upload or search an EHR, a transaction is called from the smart contract to store or search the bitmap index on the Ethereum blockchain. Hence, all these transactions are recorded with the transaction hash, sender’s account and contract’s address.

## 5 Security analysis and performance evaluation

This section assesses the security level and the performance evaluation of the proposed Blockchain-based Healthcare Management System with Two-side Verifiability (BHMV) scheme. Security analysis discusses the properties that can be satisfied in the proposed scheme for achieving the security goals. Performance evaluation tests the BHMV implementation to assess the performance-related results for system efficiency analysis. In feature comparison section, we compare the features of BHMV against the different solutions proposed in the existing literature.

### 5.1 Security analysis of the BHMV scheme

Security is the utmost important aspect when the patients are sharing their healthcare records. The confidentiality and integrity of the Electronic Health Record (EHR) need to be protected at all costs against the attack from malicious parties. Hence, we inspect the security level of BHMV based on the three security goals and the corresponding proof of work.

#### 5.1.1 Security goal 1: EHR confidentiality

The cloud storage and server should not gain any knowledge from the outsourced EHR on their premises. Apart from that, suppose an adversary breaches the cloud storage and obtains the EHR, the adversary should not be able to read or carry out a known ciphertext attack to obtain any knowledge from the EHR.

In the BHMV scheme, symmetric encryption is used to encrypt all the EHRs before outsourcing them to third-party online storage. The EHRs are encrypted with a user-defined secret key using a 256-bit Advanced Encryption Standard Cipher-Block Chaining (AES-CBC) encryption algorithm. As a result, the adversary cannot decrypt and read the EHR ciphertext without the EHR owner’s symmetric key. Additionally, the initialisation vector used for encryption can hide the ciphertext pattern by adding the random factor to each block of the cipher. Therefore, encrypting an identical block of plaintext will not result in the identical ciphertext that may reveal the ciphertext pattern to the adversary.

Notice that the entries of the bitmap index are computed with a different secret key. The separate keys prevent any similarities between the encrypted keyword matrix and the EHR ciphertext. The document identifiers in the bitmap index are created from the file hash values, preventing leaking any information about the document. The search of the encrypted keyword is done in the blockchain—a separate network from the database where the EHR ciphertext is stored. As a result, the cloud service provider would not gain any information between the search token and search result. Finally, the underlying communication channel is assumed to have additional protection in place under the protocol level, such as SSL/TLS. This will prevent adversaries from obtaining any link between the searches and the search results from different sessions, even if they were aware (e.g., revoked users) of the past searched keyword and search results. As a result, the BHMV scheme should be secure against adaptively chosen keyword attacks. Under the circumstances, the confidentiality of the outsourced EHR is safeguarded against the adversary.

#### 5.1.2 Security goal 2: Two-side verifiability

Consider an intermediate party intersects and modifies the search token *T* or the search result *R* transmitted from the user-side or the server-side. The man-in-the-middle adversary tampers with the message and signs a new Elliptic Curve Digital Signature Algorithm (ECDSA) digital signature based on the hash value of the modified message. Upon receiving the modified message, the digital signature will fail the verification test and the message will be discarded.

In the EHR searching phase, the search request from the user and the search result from the server are needed to be verified and authenticated with an ECDSA signature. If the signer’s ECDSA public key is not present in the access list on the blockchain, the recovered ECDSA signature will automatically fail the verification process. Moreover, if the hash function *H*_*ECDSA*_ used is collision-resistant, the adversary is unable to forge an ECDSA digital signature under a chosen message attack. Therefore, the adversary cannot carry out a man-in-the-middle attack for spoofing as a legitimate user due to the two-side verifiability.

#### 5.1.3 Security goal 3: Storage immutability

BHMV provides a durable medium for storing the EHR safely even in the outsourced environment. Assuming an adversary gains access and tries to modify the EHR on the Interplanetary File System (IPFS) storage, the malicious modification attempt of the EHR can be easily identified and prevented. The EHR index used for searching is also tamper-proof from any malicious party who tries to breach the search result accuracy by tampering with the EHR index.

Given the hash function *H*_*IPFS*_ used by IPFS storage, any minor modification in the EHR will result in an extensively new hash value. By comparing the modified EHR hash value to the original EHR hash value stored on the Ethereum blockchain, the attempt of EHR modification can be easily identified. In addition, the hash function used is collision-resistant, in which it is infeasible for the adversary to compute the same hash value with the modified *EHR*′. Given *EHR* ≠ *EHR*′, it is computationally infeasible to find *H*(*EHR*) = *H*(*EHR*′). Since the index of the EHR is stored on the distributed ledger of the Ethereum blockchain, the index storage is inherently supporting tamper-proof and non-repudiation properties from the characteristics of the blockchain storage. Consequently, the data storage of the EHR and its corresponding index satisfies the storage immutability property.

### 5.2 Performance evaluation of BHMV modules

Personal healthcare records are considered as highly sensitive data, the availability of EHR datasets in public is limited due to the permission required from relevant ethical bodies. Due to such constraints, this research only uses publicly available dummy EHR datasets that are artificially generated from the EMRBOTS website [25]. Inside the obtained EHR datasets, there are a total of 36,144 records in the dataset. The test EHR dataset consists of 4 columns of data, namely PatientID, AdmissionID, PrimaryDiagnosisCode and PrimaryDiagnosisDescription. PatientID is the EHR’s owner identity for claiming the ownership of the particular EHR. AdmissionID acts as the identifier for each different session of admission by a patient. PrimaryDiagnosisCode and PrimaryDiagnosisDescription are the results of the hospital’s diagnosis test on the particular patient. Some examples of the dummy datasets are provided in Table 3.

**Table 3:** Illustration of the patient’s EHRs.

PatientID	AdmissionID	PrimaryDiagnosisCode	PrimaryDiagnosisDescription
D8FD	5777	E09.42	Chemical induced diabetes
C8FF	3422	O29.123	Anaesthesia cardiac failure
A24O	4222	M84.561	Pathological fracture tibia

#### 5.2.1 EHR encryption efficiency

**Fig 5:**
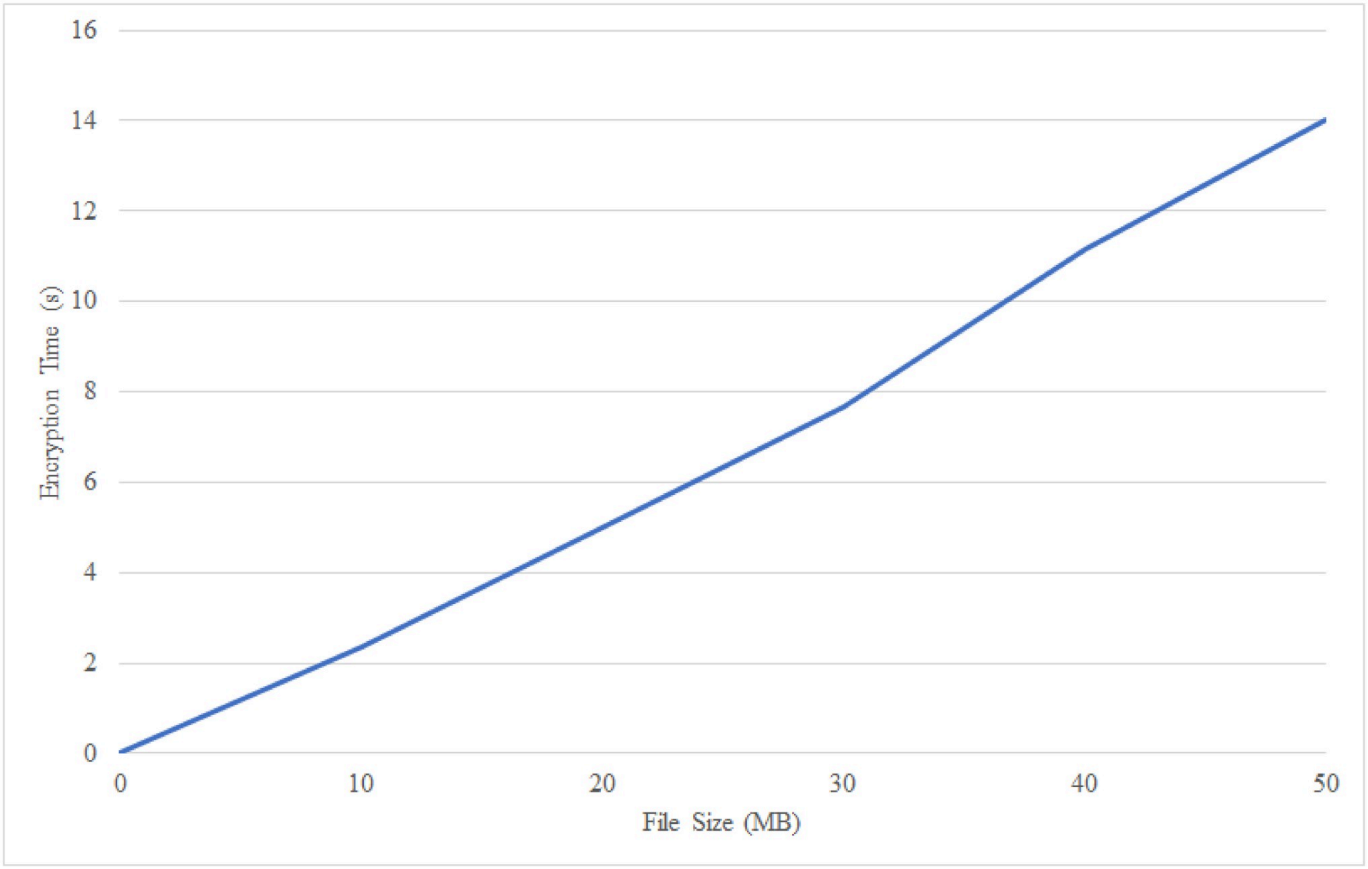
EHR encryption time based on different file sizes.

We tested the implemented system to determine the efficiency of the propose searchable encryption scheme. Fig 5 depicts the results obtained from encrypting different sizes of EHR. In general, the size of a patient’s EHR will increase over time as more diagnosis or treatment are carried out. For illustration, medical screening generates a large amount of data, such as, the output of the images from X-Ray, CT-Scan, and Ultrasonic Imaging. Therefore, this performance metric can reflect and simulate the real-world situation of EHR documentation. The result in Fig 5 shows a direct proportional of encryption time towards the EHR’s size in megabytes (MB). For example, notice that a 10 MB file requires less than 3s of encryption time. In contrast, a 50 MB file requires about 14s of encryption time. Therefore, the curve is almost linear. BHMV encryption scheme deploys an efficient AES-CBC encryption mode that can support encryption for a larger to medium size database without exponentially increasing the encryption time.

**Fig 6:**
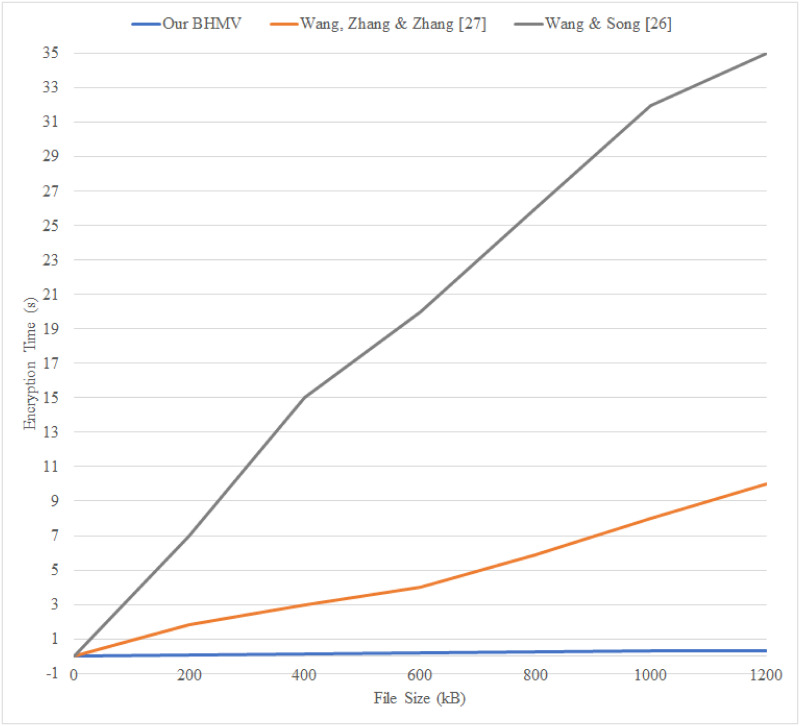
Encryption efficiency comparison.

Fig 6 demonstrates the comparison of encryption time between the schemes proposed by Wang, Zhang and Zhang [27], Wang and Song [26], and our scheme. The x-axis indicates the file size in kilobytes (KB), while the y-axis indicates the encryption time in seconds (s). Since the comparing schemes are not re-implemented, the results are referred to and obtained from the original research paper. The size of the file subject used for the comparison is limited to 1200 kilobytes (≈ 1.2 MB) to match the analysis of the similar file sizes that are used in the above two schemes. The comparison indicates that the encryption algorithm used in BHMV outperforms the other schemes in terms of efficiency. That is, the encryption time does not increment as much as the compared schemes when the file size is getting larger. Hence, our proposed solution provides a faster overall encryption performance that is future-proof and practical to be implemented in the large-scale EHR storage and sharing system.

The encryption algorithms used in both this work and the work of Wang, Zhang and Zhang use the AES encryption algorithm. The results show that BHMV has comparatively better performance; we believe this could be due to several factors, including the implementation environment and the modes of operation of the cipher. For reference, the system implementations of the comparing schemes were implemented in a computing environment with an Intel Core i5-7200U processor, 4 gigabytes (GBs) of random-access memory (RAM). The Java programming language was used for the implementation of these two schemes. In contrast, our system was implemented using JavaScript; the computing environment details of our implementation are provided in Section 4.7. Therefore, the result is partially subject to the CPU parameters, programming language and encryption algorithm variables. Additionally, the performance of both our scheme and the scheme of Wang, Zhang and Zhang is significantly high compared to the scheme of Wang and Song, especially when the file size is large. This is because Wang and Song’s approach uses an attribute-based encryption (ABE) algorithm that is computationally more expensive and requires a longer encryption time. This comparison also shows the general performance advantage of using symmetric cipher compared to asymmetric cipher such as the ABE algorithm.

#### 5.2.2 EHR indexing efficiency

**Fig 7:**
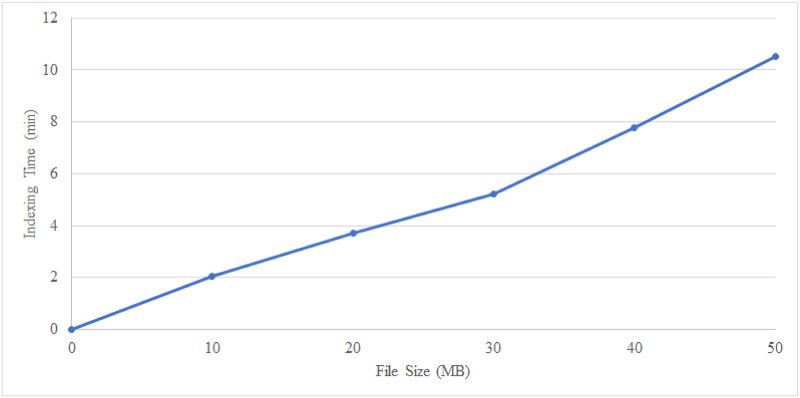
EHR indexing time based on different file sizes.

The performance recorded from the execution of the index builder module is shown in Fig 7. Index building requires a longer time to process as the index encryption module also involves the construction of the bitmap index table. The results obtained indicate an approximate linear relationship between the file size (MB) and indexing time (min), showing that the bitmap index method in our BHMV scheme is feasible to implement without incurring substantial performance penalty.

#### 5.2.3 Features comparison with existing schemes

We compare the functionalities of our proposed scheme against the existing Blockchain-based cloud storage solutions. The comparison details provided in Table 4 includes the following features: encryption, keyword search, server-side verifiability, user-side verifiability, storage integrity, dynamic update. According to the evaluation in Table 4, about half of the studied schemes do not support EHR encryption during the document pre-processing phase. The security in these schemes solely relies on blockchain, which is insufficient to protect the confidentiality. In terms of searchability, the schemes proposed by Al Asad et al. and Parameswari et al. [7, 8] did not specifically address the searchability. Meanwhile, the scheme proposed by Chelladurai and Pandian [9] can search the EHR base on a patientID. The result returned is the entire collection of the EHR owned by the corresponding patient. In other words, the EHR stored in the scheme does not support keyword search. As a result, this approach incurs high transmission overhead due to the retrieval of unrelated EHR in every read attempt. Additionally, the user needs to decrypt all the EHR ciphertext every time in order to identify the searched EHR. This application is inefficient and less desirable due to the unnecessary performance overhead and extra time required for decrypting all the EHR.

**Table 4:** Blockchain-based healthcare management system—Key features comparison (✔: Supported; ✘: Not supported).

Ref.	Encryption	KW Search	Server-side Verifiability	User-side Verifiability	Storage Integrity	Dynamic Update
[26]	✔	✔	✘	✘	✔	✘
[4]	✔	✔	✘	✘	✔	✘
[27]	✔	✔	✔	✔	✔	✘
[3]	✘	✔	✘	✘	✔	✘
[7]	✘	✘	✘	✘	✔	✘
[8]	✘	✘	✘	✘	✔	✘
[9]	✘	✘	✘	✘	✔	✔
BHMV	✔	✔	✔	✔	✔	✔

All of the schemes support a certain level of user-side verifiability in terms of the EHR retrieved from the third-party storage. However, the verification is solely based on the assurance of integrity provided by the hash value from the blockchain. Man-in-the-middle or spoofing attempt is still plausible to be deployed to deceive the user. Our proposed scheme, BHMV mitigates the issue by adding another layer of security using the ECDSA signature. Both server-side and user-side can verify the message they received from each other.

Even though the schemes proposed by Al Asad et al. and Parameswari et al. [7, 8] support EHR storage integrity, the approaches are relatively inefficient as the whole EHR is encrypted and stored on the blockchain. This approach will incur high transaction fees due to the limited block size on blockchain distributed ledger. Consequently, a big chunk of data will be split into discrete blocks and transacted separately, incurring a high amount of transaction fee for the user. Contrary to this, in our proposed scheme, the EHR ciphertext is stored in the Interplanetary File System (IPFS), and only the encrypted index is stored in the blockchain.

Most of the studied schemes do not address the support for dynamic EHR update. In the scheme proposed by Chelladurai and Pandian [9], EHR updates functionality is considered and mentioned. However, this scheme lacks many other important security features. The scheme proposed by Wang et al. [27] achieves most of the features specified in Table 4; however, it does not support dynamic EHR update. In the proposed BHMV scheme, dynamic update is supported by the construction of bitmap index. Since the bitmap index matrix is dynamic in terms of the size, any modification or addition of the EHR does not require to rebuild the index. The bitmap index can be updated easily by creating new row and column to accommodate the newly uploaded data.

## 6 Conclusions

We proposed a Blockchain-based healthcare management system with two side verifiability (BHMV) to reduce the gap between the proposed existing solutions and real-life implementation. The proposed solution is lightweight yet secured. BHMV combines the benefits provided by blockchain technology with the cryptographic system to construct a secure and convenient platform for EHR storage and sharing.

The encryption algorithm for the proposed system uses a state-of-the-art encryption algorithm: Advanced Encryption Standard. For implementing the two-side verifiability feature, Elliptic Curve Digital Signature Algorithm (ECDSA) is used to sign and verify the signature’s authenticity. ECDSA verification is required during the EHR searching and access phases. A private blockchain network is implemented in the BHMV scheme to eliminate a Trusted Third Party (TTP) requirement. The construction of the bitmap index allows for a dynamic searchable symmetric encryption in which the index can be updated easily by changing the tuple’s value of a particular keyword. The process of splitting the storage by storing only the bitmap index on the blockchain is aimed to reduce the transaction fee that may incur if we include large datasets, such as the whole EHR on the blockchain ledger. The results obtained from the prototype developed are promising with efficient indexing and encryption rate against large EHR files. The high-efficiency performance complies with the security goals of confidentiality, immutability and two-side verifiability.

To conclude, blockchain application in the healthcare industry is plausible and practical. It natively support various security requirements. We have incorporated the notions of searchable symmetric encryption (SSE), ECDSA digital signature, and blockchain technology to implement a secure yet practical prototype to be deployed in the healthcare industry.
